# Topoisomerase inhibitors in cervical cancer: mechanistic insights and therapeutic strategies

**DOI:** 10.1186/s10020-026-01446-z

**Published:** 2026-03-04

**Authors:** Yashaswini Reddy, Padmini Pai, Ipshita Das, Shruthi Nayak, Neil Viren D’Souza, Manasa Gangadhar Shetty, Babitha Kampa Sundara

**Affiliations:** 1 Department of Biophysics, Manipal School of Life Sciences, Manipal Academy of Higher Education, Manipal, Karnataka 576104 India; 2 Manipal School of Life Sciences, Manipal Academy of Higher Education, Manipal, Karnataka 576104 India

**Keywords:** Cervical cancer, Topoisomerase I and II, Inhibitors, Treatment efficacy, Toxicity, Dosage

## Abstract

Cervical cancer represents a critical global health burden, particularly among women in countries with limited healthcare infrastructure, where it accounts for a substantial proportion of cancer-related morbidity and mortality. It remains highly prevalent in developing regions. Most cervical cancers are squamous cell carcinomas originating from the cervix lining cells, mainly because of chronic infection with high-risk human papillomaviruses (HPV) types. Additionally, the likelihood of infection is enhanced by behaviours include early sexual activity, multiple partners, smoking, and immunosuppression. Topoisomerases, vital enzymes for DNA replication and transcription are crucial for therapeutic intervention in cancer. Inhibitors of Topoisomerase I and II, such as camptothecin derivatives (topotecan, irinotecan) and drugs like etoposide and daunorubicin, have shown effectiveness in treating cancers, including cervical cancer, by stabilizing topoisomerase-DNA complexes and inducing lethal DNA strand breaks. However, traditional treatments like chemotherapy and radiation often have high toxicity and limited survival benefits. The development of new topoisomerase inhibitors with better efficacy and safety is essential. Advances in tumour biology have led to multi targeting drugs that target multiple cancer cells survival pathways, offering a more comprehensive anti-cancer effect. This review explores the application of topoisomerase inhibitors in advanced and recurrent stages of cervical cancer, both as standalone agents and in combination therapies. Combining topoisomerase inhibitors with other treatment options has been used as a strategy to improve therapeutic outcomes. Additionally new therapeutic strategies like nanoformulations, antibody drug conjugates, and biomarker-based therapies lead to effective therapeutic outcomes in cervical cancer treatment. Ongoing research and development in this area are crucial for enhancing cervical cancer treatment.

**Graphical Abstract:**

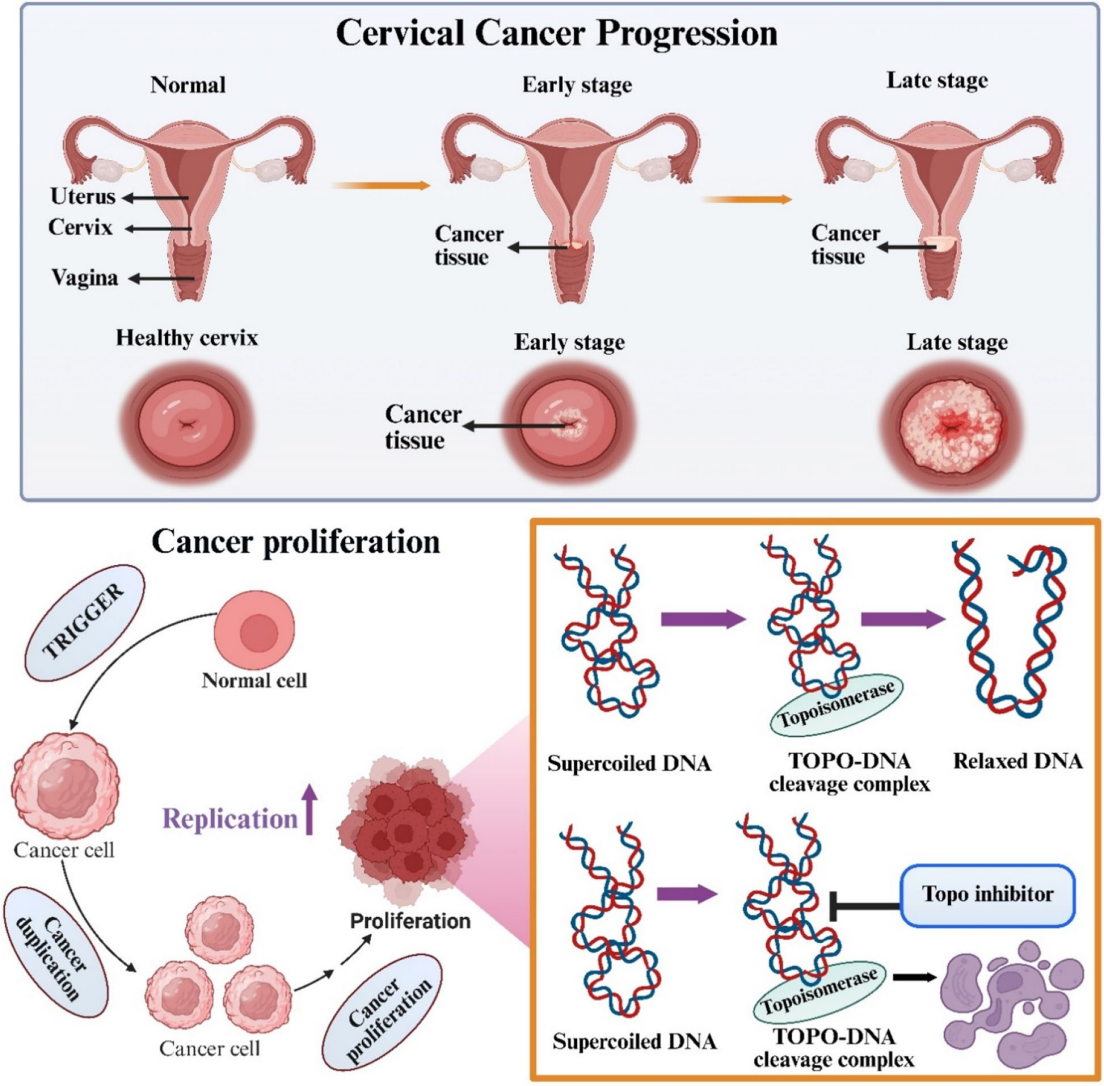

## Introduction

Cervical cancer is one of the critical global health burden, with approximately 660,000 new cases reported in 2022, making it as the fourth most prevalent cancer affecting women. World Health Organization (WHO) estimates indicate that nearly 94% of the 350,000 deaths linked to cervical cancer occurred in countries with limited healthcare infrastructure (https://www.who.int/news-room/fact-sheets/detail/cervical-cancern.d.). In 2018, the global incidence was around 570,000 cases, leading to 311,000 deaths. The global incidence rate was estimated at 13.1 per 100,000 women, though this figure varies widely across different regions. Notably, cervical cancer is particularly prevalent in developing countries, where three-quarters of cases occur. In African region, cervical cancer was the accounted for the highest number of neoplasm-related mortality among women in 2018, with occurrence rates indicating that nearly 6.5% of women are projected to develop the disease before the age of 75 (Arbyn et al. 2020). Additionally, China and India together contributed to over one-third of the total global cervical cancerous burden. Specifically, China reporting 106,000 cases and 48,000 deaths, and India 97,000 cases and 60,000 deaths. Worldwide, the mean age at which diagnosis occurs is projected to be around 53 years, with the average age at death is 59 years. Among women aged 20–39 years, cervical cancer remains the second most common cause of cancer mortality, resulting in approximately 10 premature deaths per week in this age group (Buskwofie et al. 2020). This indicates that there is a need for increased screening, vaccination, and treatment options mainly in regions with high incidence and death rates.

Cervical cancer occurs from the cells lining the cervix, with squamous cell carcinomas arising from exocervical cells and adenocarcinomas originating from mucus-producing endocervical gland cells. Most cervical cancer are squamous cell carcinomas, starts from the “transformation zone”, where the exocervix and endocervix converge (Psilopatis et al. 2023). The predominant cause of cervical cancer is enduring infection with cancer-inducing, high-risk strains of Human Papillomavirus (HPV) (Burmeister et al. 2025). Additional contributing factors include those are correlated with the exposure to mutagens, hormonal influences, immune dysfunction and sexual acquisition of HPV. Genetic predisposition appears to have a limited involvement in development of cervical cancer, as indicated by existing studies. Major causative determinants comprise onset of sexual intercourse at a young age, having numerous sexual partners, contact with additional sexually transmitted pathogens, tobacco consumption, use of birth control pills, infection with human immunodeficiency virus (HIV), and treatment involving immune-suppressing medications (Zhang et al. 2020). Socioeconomic factors also play a significant role, as limited access to cancer screening services and declined compliance with screening appointment are more prevalently observed among women of lower socioeconomic and educational backgrounds. These disparities are particularly noted among African American, American Indian, and Hispanic women compared to Caucasian women in the United States. These particular socioeconomic barriers adversely affect HPV vaccine uptake in these populations, impaired by the vaccine's high cost (Sathiyaseelan et al. 2025).

**Fig. 1:**
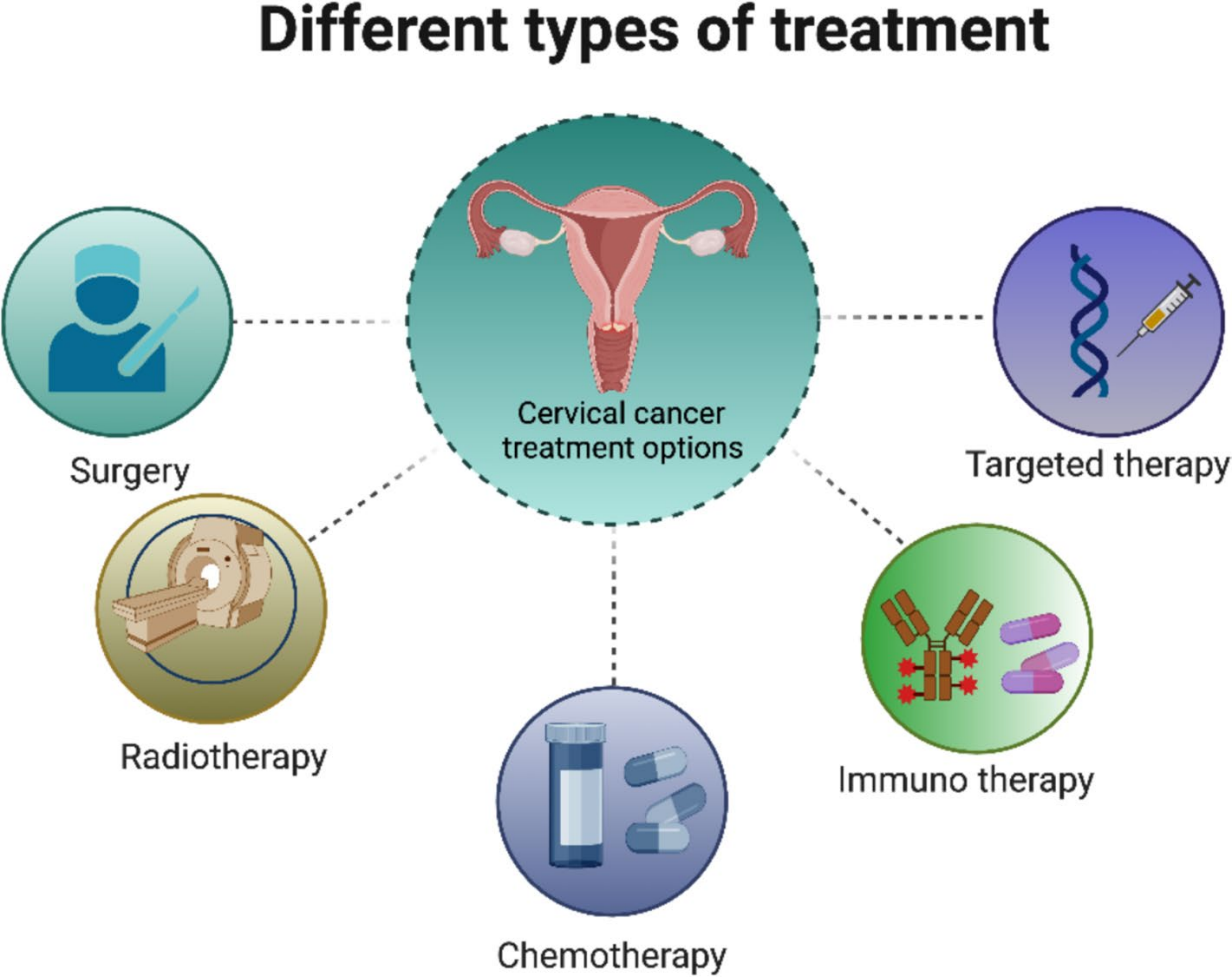
Different types of treatment options in cervical cancer which includes surgery, radiotherapy, chemotherapy, immune therapy and targeted therapy (Created using BioRender)

The management of cervical cancer varies widely depending on the stage at diagnosis, with early-stage disease amenable to localized treatments and advanced or recurrent disease requiring systemic approaches. The current treatment modalities for cervical cancer, encompassing surgical interventions, radiation therapy, chemotherapy, targeted therapy, immunotherapy, and novel therapeutic strategies (Fig. 1) (Burmeister et al. 2022). These different treatment modalities underscore the need for targeted approaches that disrupt cancer cell proliferation. Among such approaches, the inhibition of DNA topoisomerases has emerged as an important strategy, given their fundamental role in maintaining DNA topology during replication and transcription.

This paper is structured as a mechanistic and therapeutic narrative review, encompassing the molecular mechanisms, translational insights, and both pre-clinical and clinical studies of topoisomerases in the treatment of cervical cancer. Its scope is to provide a comprehensive overview of the mechanistic roles of Topoisomerase I and II and their dysregulation in cervical cancer. Additionally, it discusses about the preclinical insights with clinical observations and explain the reasons behind discrepancies between experimental success and clinical outcomes.

### Topoisomerases

DNA topoisomerases are ubiquitous enzymes essential across all domains of life, addressing the various topological challenges posed by the extensive human DNA double helix, which spans approximately 3 × 10^9^ base pairs. This DNA must be intricately folded, bent, and compacted within the cell nucleus while remaining accessible for transcription and replication by RNA and DNA polymerases. Each human cell also harbors between 100 and 1,000 copies of circular mitochondrial DNA (mtDNA), approximately 16,000 base pairs in length, alongside abundant long and folded RNAs distributed throughout all subcellular compartments (Pommier et al. 2022).

Topoisomerases play critical roles in cellular processes such as chromosome condensation, segregation, and gene expression (Hsiang et al. 1989). Although all topoisomerases perform the fundamental task of interconverting the topological states of DNA, the mechanisms employed vary among different enzyme classes (McKie et al. 2021). During transcription and replication, the unwinding of double-stranded DNA forms left-handed (positive) and right-handed (negative) supercoils on both sides of the unwound region. Positive supercoiling increases the tension and compacts the DNA structure. Positive supercoiling increases DNA tension, restricting strand unwinding and obstructing the process of DNA synthesis. In absences of topoisomerases, the accumulation of positive supercoils and DNA entanglement would hinder, and eventually halt, both transcription and replication (Seo 2015).

A unifying feature of all topoisomerases is establishment of a covalent DNA-topoisomerase intermediate. This process involves, tyrosine residue at the enzyme’s active site of topoisomerase carries out a nucleophilic attack on the DNA backbone, producing a phosphotyrosyl bond with a phosphate moiety. Topoisomerases are categorized into type I and type II based on their activity; type I topoisomerases mediate the cleavage and subsequent rejoining of single-stranded (ss) DNA breaks, while type II topoisomerases manage double-stranded (ds) DNA breaks (McKie et al. 2021).

### Toposiomerase I

A single copy gene on chromosome 20q12-13.2 expresses the 100 kDa monomeric protein Topoisomerase I (Topo I), which requires phosphorylation to function catalytically (Sharma et al. 2024). Topo I enzymes are crucial role in catalyzing the relaxation of supercoiled DNA during necessary step for key intracellular activities including chromatin condensation, recombination, replication plus transcription. Unlike most enzymes, Topo I enzymes, apart from reverse gyrase, do not require adenosine triphosphate (ATP) for their catalytic activity. They are classified into two main types based on their structure characteristics and mechanism of action: Type IA and Type IB.

Type IA topoisomerases, which include bacterial and archaeal Topo I, topoisomerase III, and reverse gyrase, operate by forming a covalent linkage between the 5′ end of the cleaved DNA strand and a catalytic tyrosine residue within the enzyme. In order to relax negative supercoils, these enzymes require Mg^2+^. In contrast, Type IB topoisomerases, such as eukaryotic Topo I and Topoisomerase V, use a covalent intermediate with the 3′ end of the cleaved DNA strand through a catalytic tyrosine residue. Type IB enzymes can relax both positive and negative supercoils without requiring Mg^2+^. Furthermore, they have little structural and sequence homology with other topoisomerase groups, emphasising their functional difference from Type IA enzymes (Buzun et al. 2020).

Human DNA topoisomerase IB (hTopIB) is a 100-kDa protein that relaxes DNA supercoils by cleaving a single strand of DNA, allowing it to rotate, and re-ligating it. The catalytic tyrosine residue (Tyr723) initiates the process through a nucleophilic attack on the DNA backbone. This mechanism causes the cleavage of one DNA strand and the development of a covalent bond between Tyr723 hydroxyl group and the DNA’s 3′-phosphate resulting in a transient cleavage complex. After the DNA’s linking number is altered, the 5′-hydroxyl end initiates a second nucleophilic attack, reconnecting the DNA strands and releasing the enzyme (Soren et al. 2020).

### Topoisomerase II

Topoisomerase II (Topo II) has two forms: the 170-kDa Topo IIα and the 180-kDa Topo IIβ. Despite their high resemblance, these forms are encoded by distinct genes. Topo II enzymes introduce double-stranded breaks in DNA through an ATP-dependent process, allowing one double-stranded DNA molecule to pass through another (McLeod et al. 1994).

In eukaryotes, II α enzymes relax both positive and negative supercoils and perform important roles in decatenation and unknotting of DNA helices. Bacterial type IIα enzymes, including DNA gyrase and topoisomerase IV (Topo IV), help resolve DNA topological difficulties through decatenation and strand passage activities (Evoli et al. 2024). Topo IIβ causes transitory double-strand breaks during transcription, changing DNA’s topology. Topo IIβ’s association with the DNA strand plays an important role in regulating gene transcription. The catalytic cycle of Topo II manipulates DNA topology in an ATP-dependent way.

The process begins when the Topo II enzyme recognizes a DNA target (G segment) and causes breaks in both strands, resulting in a DNA breakage complex. The ATPase domain at the N-terminus of the cleavage complex is located above the DNA breakage core region. When two ATP molecules bind together, the complex undergoes a structural shift that allows a second double-helical DNA segment (T segment) to pass through a momentary break in the G segment. The transfer of strands reduces the DNA supercoiling. The break in the G segment is then sealed, and the ATP molecules are broken down by hydrolysis (Vejpongsa And Yeh 2014).

### Inhibitors of topoisomerases

Topoisomerase I (Topo I) and topoisomerase II (Topo II) inhibitors exert anticancer effects through mechanistically distinct but biologically interrelated ways of DNA damage induction, resulting in varying clinical efficacy and toxicity profiles. Topo I inhibitors, such as irinotecan and topotecan, stabilize the Topo I-DNA cleavage complex after single-strand breaks to prevent religation, resulting in replication fork collisions primarily during S-phase, whereas Topo II inhibitors like etoposide and doxorubicin freeze the Topo II homodimer after double-strand DNA cleavage, blocking strand passage and re-ligation to induce breaks throughout the cell cycle (Yakkala et al. 2023).

In terms of clinical efficacy, Topo I inhibitors outperforms in colorectal cancer with irinotecan extending median survival by 2.2 months, and topotecan yielding 20–30% response rates in ovarian cancer, while Topo II inhibitors achieve 70–80% complete remission in Acute Myeloid Leukaemia induction with daunorubicin and over 60% 5-year survival in aggressive lymphomas through doxorubicin (Hartmann and Lipp 2006).

Safety profiles differ markedly, as Topo I inhibitors predominantly cause severe diarrhea (grade 3–4 in 20–30% of irinotecan cases) and neutropenia (40% with topotecan) with lower cardiotoxicity risks compared to Topo II inhibitors' higher cardiotoxicity (5% CHF at doxorubicin doses > 450 mg/m^2^), secondary leukemias (1–2% etoposide risk peaking 2–3 years post-treatment), and alopecia (80–90%) (Pommier 2009; Nitiss 2009). Currently, Topo I inhibitors serve as standards in metastatic colorectal cancer and small cell lung cancer while Topo II inhibitors are frontline in pediatric Acute Lymphoblastic Leukemia and Hodgkin lymphoma (Bondarev et al. 2024; Schöffski et al. 2024) Fig. 2. Represents the general mechanism of action of topoisomerase and topoisomerase inhibitors.

**Fig. 2:**
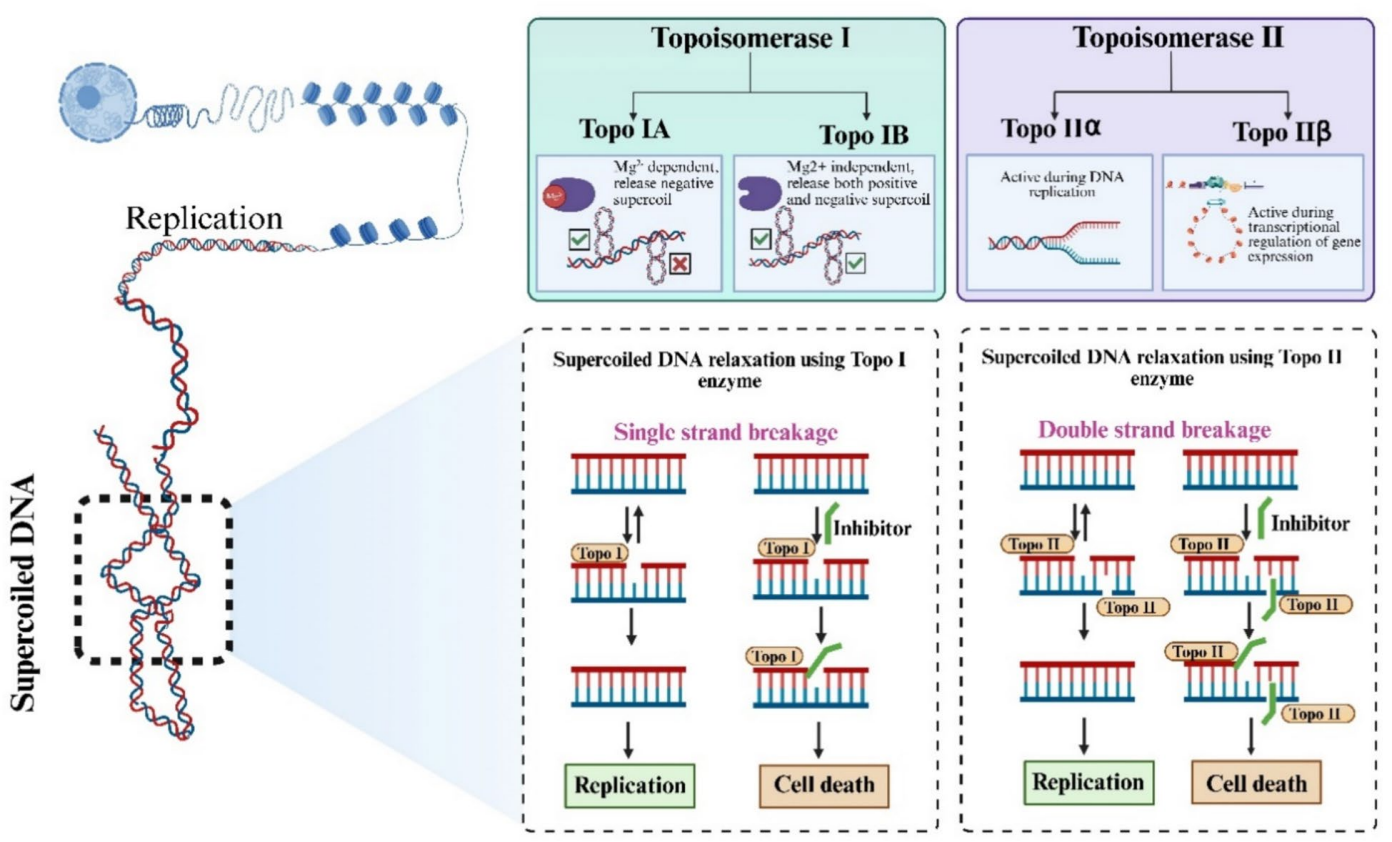
General mechanism of action of Topoisomerase and Topoisomerase inhibitors. Topo I introduce a temporary single-strand break and allows controlled rotation of DNA without requiring ATP, whereas Topo II creates a transient double-strand break, passes another DNA segment through it, and religates the strands in an ATP-dependent manner. Topoisomerase inhibitors act by interfering with this catalytic cycle, primarily by stabilizing the enzyme–DNA cleavage complex and preventing religation. This leads to accumulation of DNA breaks, replication fork collapse, activation of DNA damage response pathways, and ultimately apoptosis, particularly in rapidly proliferating cancer cells (Created using BioRender)

Camptothecin (CPT) is a plant alkaloid originally identified from the Chinese tree, *Camptotheca acuminata*. Shortly after its discovery, CPT was found to inhibit Topo I by acting as a Topo I poison. Currently, two water-soluble camptothecin derivatives, topotecan and irinotecan, have been approved by the FDA for intravenous administration (Pommier 2009).

Topo II-targeted drugs are broadly classified into two major groups. The first group includes direct topoisomerase II toxins, with many clinically active agents falling into this category (e.g., etoposide, daunorubicin, mitoxantrone). These agents increase the levels of topoisomerase II-DNA covalent complexes, effectively hindering transcription and replication processes. Topo II inhibitors induce single or double stranded nicks in DNA, disrupting both DNA replication during the cell cycle and the function of RNA polymerase.

The second class consists of catalytic inhibitors of Topo II. These molecules act by blocking the critical catalytic function of Topo II. Among the most notable members of this group are the bisdioxopiperazines, such as dexrazoxane (ICRF-187), along with experimental compounds Razoxane (ICRF-159) and Sobuzoxane (MST-16). Even though these medications show limited effectiveness against tumors, their main therapeutic use lies in minimizing the cardio-related toxicity caused by anthracyclines (Economides et al. 2019). Tables 1 and 2 represents the list of FDA approved Topo I and II inhibitors.

**Table 1:** List of FDA approved Topo I Inhibitors

FDA approved Topo I inhibitors	Structures
Topotecan	
Irinotecan

**Table 2:** List of FDA approved TopoII Inhibitors

FDA approved Topo II inhibitors	Structures
Doxorubicin	
Daunorubicin	
Idarubicin	
Mitoxantrone	
Etoposide	
Teniposide	

### HPV and topoisomerases

In HPV-positive cervical cancer, the virus attacks host topoisomerases to support its life cycle. The viral oncoproteins E6 and E7 drive the upregulation of Topo Iα and Topo IIIβ, which bind the viral upstream regulatory region (URR) to ensure efficient genome replication and gene expression, while activating DNA damage and inflammatory pathways (Vats et al. 2026). Meanwhile, TopoIIβ helps relieve torsional stress during transcription and replication and, together with CTCF (CCCTC-binding factor)/cohesin complexes, orchestrates viral transcription and replication, a process that can influence resistance to topoisomerase inhibitors (Kaminski et al. 2021). Targeting these enzymes with inhibitors such as camptothecin, especially when combined with TDP1 (Tyrosyl-DNA Phosphodiesterase 1)/PARP1 (Poly (ADP-ribose) polymerase −1) blockade, can synergistically suppress high-risk HPV replication (Toots et al. 2017). Beyond viral replication, cervical cancer cells themselves exploit topoisomerases: Topo IIα and CENPF (Centromere Protein F) emerge as synergistic master regulators, driving proliferation and metastasis, often in the context of p53 or Rb1 alterations, and represent promising targets for topoisomerase-directed therapies like etoposide (Yu et al. 2020). Together, these findings highlight how HPV oncoproteins and topoisomerases intertwine to promote viral persistence and cervical cancer progression, offering multiple therapeutic opportunities.

Inhibitors of topoisomerases emerged as a therapeutic strategy in controlling cervical cancer, targeting important enzymes involved in DNA replication. Blocking these enzymes interferes with DNA synthesis and cellular proliferation, eventually resulting in the elimination of cancer cells. This article examines the involvement of topoisomerase inhibitors in cervical cancer treatment, exploring their mode of action, clinical applications, and efficacy.

### Topoisomerase I inhibitors in pre-clinical studies

Balat and Verschraegen (1995) reported the anticancer activity of pyrazoloacridine derivatives, particularly the 9-methoxy acridine analogue, which act as DNA intercalators and induce single- and double-strand DNA breaks, thereby disrupting DNA and RNA biosynthesis (Balat and Verschraegen 1995). SN-38 treatment on HeLa and SiHa cells resulted in a dose- and time-dependent increase in p53 and p21 expression, leading to growth inhibition. Akt cDNA transfection reduced SN-38-induced apoptosis, indicating that inhibition of the Akt pathway enhances SN-38 cytotoxicity in cervical cancer cells (Liu et al. 2009). Van et al. demonstrated that 3-arylisoquinolinamine derivatives possess topoisomerase inhibitory activity and suppress cancer cell proliferation. Compound 1, a piperazine-substituted 3-arylisoquinolinamine, induced G0/G1 phase cell-cycle arrest and exhibited selective inhibition of Topo I over Topo II, which was further supported by molecular docking studies (Van et al. 2014). Hydantoin and thiohydantoin derivatives exhibit broad anticancer potential. Majumdar et al. (2015) identified several 3,5-disubstituted hydantoins with selective cytotoxicity. Among them, Compound 2 showed potent inhibition of human DNA Topo I and strong cytotoxicity against HeLa and MCF-7 cells. The presence of a methoxy group on the pyridine ring and substitution at the C5 position of the thiophene ring were key contributors to the enhanced anticancer activity of Compound 2 (Majumdar et al. 2015). Hydroxycamptothecin (HCPT), a Topo I inhibitor, was studied by Cheng et al. (2015) induced autophagy in cervical cancer cells through downregulation of miR-30a. miR-30a binds to the 3′UTR of Beclin-1, and its overexpression suppressed HCPT-induced autophagy, elucidating the molecular mechanism of HCPT action (Cheng et al. 2015). Kovvuri et al. synthesized β-carboline-bisindole compounds that inhibit Topo I and interact with DNA. Compounds 3a and 3b displayed significant antiproliferative activity against HeLa cells, with IC₅₀ values of 2.54 ± 0.91 µM and 3.25 ± 1.77 µM, respectively (Kovvuri et al. 2018). Nagaraju et al. (2019) synthesized pyrazole-linked benzothiazole-β-naphthol derivatives with selective cytotoxicity toward HeLa cells. Compounds 4a, 4b, and 4c induced G2/M phase arrest and exhibited IC₅₀ values of 5.20 µM, 5.54 µM, and 4.63 µM, respectively. Electron-donating substituents enhanced cytotoxicity, while electron-withdrawing groups reduced activity (Nagaraju et al. 2019). Lee et al. (2019) evaluated CDK-602, a synthetic camptothecin analogue, which induced apoptosis, increased PARP, cleaved PARP, BAX, and phosphorylated p53 expression, suppressed invasion, and caused G2/M phase arrest in cervical cancer cells. In vivo xenograft studies demonstrated significant tumor volume reduction following CDK-602 treatment (Lee et al. 2019). Haider et al. (2019) synthesized 9-(2-(1-aryl ethylidene) hydrazine) acridine derivatives. Compounds 5b, 5 d, and 5e showed strong antiproliferative effects against HeLa cells by inducing DNA damage, inhibiting Topo I, and causing S-phase arrest, with IC₅₀ values of 20.97 ± 0.55 µM, 18.89 ± 0.61 µM, and 20.66 ± 0.60 µM, respectively (Haider et al. 2019). Zhou et al. (2022) developed 6H-chromeno[3,4-b] quinoline derivatives based on boeravinones. Compound 6 exhibited the highest cytotoxicity against HeLa cells (IC₅₀ = 4.37 µM). Compounds 6a and 6b showed IC₅₀ values of 7.75 µM and 13.1 µM, respectively, along with 81% Topo I inhibition at 2.5 µM and 84% inhibition at 5.0 µM, supporting their potential as non-camptothecin Topo I inhibitors. Table 3 represents the list of Topo I inhibitors in pre-clinical studies (Zhou et al. 2022).

**Table 3:** Cytotoxicity and enzyme inhibition profile of TOPO I inhibitors

TOPO I Inhibitors	MTT assay IC_50_ values	Enzyme inhibition/drug concentration	Cell lines	References
	-	73.0% at 100 µM 0.6% at 20 µM	HeLa	(Van et al. 2014)
	1.17 μM	10.08 ± 0.325 μM (E	HeLa	(Majumdar et al. 2015)
	2.54 ± 0.91 μM	20 μM	HeLa	(Kovvuri et al. 2018)
	3.25 ± 1.77 μM	20 μM	HeLa	(Kovvuri et al. 2018)
	5.20 ± 1.62 μM	100 μM	HeLa	(Nagaraju et al. 2019)
	5.54 ± 0.99 μM	100 μM	HeLa	(Nagaraju et al. 2019)
	4.63 ± 1.29 μM	100 μM	HeLa	(Nagaraju et al. 2019)
	20.97 ± 0.55 μM	2 μM	HeLa	(Haider et al. 2019)
	18.89 ± 0.61 μM	2 μM	HeLa	(Haider et al. 2019)
	20.66 ± 0.60 μM	2 μM	HeLa	(Haider et al. 2019)
	7.75 μM	81% at 2.5 μM	HeLa	(Zhou et al. 2022)
	13.1 μM	84% at 5.0 μM	HeLa	(Zhou et al. 2022)

Collectively, these studies emphasize the importance of diverse topoisomerase-targeting scaffolds in cervical cancer therapy due to their ability to induce DNA damage, regulate cell-cycle arrest, and trigger apoptosis or autophagy. Compound 1 (piperazine-substituted 3-arylisoquinolinamine) showed selective Topo I inhibition with G0/G1 arrest, while Compound 2 (3,5-disubstituted hydantoin) exhibited strong Topo I inhibition and HeLa cytotoxicity. β-Carboline–bisindole compounds 3a and 3b were highly potent (IC₅₀ = 2.54 ± 0.91 and 3.25 ± 1.77 µM). Compound 6 and its analogues 6a/6b (6H-chromeno[3,4-b]quinolines) showed notable HeLa activity (IC₅₀ = 4.37, 7.75, and 13.1 µM) with high Topo I inhibition, supporting further optimization and in vivo studies.

### Topoisomerase I inhibitors in clinical trials

Topoisomerase I drugs, particularly topotecan, had been evaluated for safety and effectiveness in patients with cervical squamous cell carcinoma in a different multicenter II phase study. The drug was administered at a dose of 1.5 mg/m^2^ per day for 5 consecutive days every 4 weeks. Approximately 7.0% and 11.6%, respectively, of the 43 patients evaluated gave complete and partial answers. According to Muderspach et al. (2001), topotecan was linked to a considerable hematologic damage despite its modest effectiveness in treating advanced, recurring, and persistent cervical squamous cell carcinoma (Muderspach et al. 2001). For individuals with recurrent or persistent cervical carcinoma who were unsuitable for surgical intervention or radiotherapy as curative options, a phase II study was conducted to assess the safety and efficacy of the topoisomerase I inhibitor belotecan. The dosage of Belotecan administered in the study was 0.5 mg/m^2^/day for 5 consecutive days every 3-week cycle. Of the 16 patients included in the research, 87.5% (14 out of 16) had undergone radiation or chemotherapy before. Over the course of three weeks, belotecan was given for five days in a row. Non-hematologic toxicities were uncommon in the study, although serious side events were recorded, including anemia and neutropenia. A patient passed away from toxicity associated with their therapy. The median overall survival was 12.38 months, and no patient showed any response to belotecan. Half of the patients showed signs of disease advancement, while 12.5% exhibited no change in disease status. The study concluded that belotecan lacked efficacy as a second-line treatment for progressive or recurrent cervical cancer (Hwang et al. 2011). Table 4 represents the list of Topo I inhibitors in clinical trials.

**Table 4:** Topo I inhibitors in clinical trails

Cancer type	Drugs (dose)	Number of evaluable patients	Complete response	Partial response	Response rate	Reference
Cervical squamous cell carcinoma	Topotecan (1.5 mg/m^2)^	43	7.0%	11.6%	-	(Muderspach et al. 2001)
Recurrent or persistent cervical carcinoma	Belotecan (0.5 mg/m^2^)	16	0	0	-	(Hwang et al. 2011)

#### Limitations of Topo I inhibitors in clinical trails

Among the Topoisomerase I inhibitors evaluated, topotecan showed only modest activity, with low response rates and significant hematologic toxicity, while belotecan demonstrated poor clinical efficacy with no objective responses and notable severe toxicities. Overall, neither drug proved effective for recurrent or advanced cervical cancer, and no strong or superior compound emerged from these studies, highlighting the limited success of current Topo I inhibitors in clinical settings for cervical cancer.

### Topoisomerase II inhibitors pre-clinical studies

Cho et al. (2010) synthesized 12 tetracyclic benzoxanthone derivatives and evaluated their cytotoxicity, topoisomerase inhibition, and DNA crosslinking activity. Compound 7 showed the highest Topo II inhibition (60.1% at 100 μM) and significant cytotoxicity against HeLa cells (IC₅₀ = 2.19 ± 0.70 μM), attributed to its tetracyclic scaffold and functional group–mediated interactions with DNA–protein complexes (Cho et al. 2010). Iacopetta et al. evaluated four 1,4-dimethylcarbazole derivatives, ellipticine analogues, for selective inhibition of human DNA topoisomerase II while sparing normal cells. Compounds 8 and 9 showed strong hTopo II inhibition, good thermal stability, and effective cytotoxicity against HeLa cells, with IC₅₀ values of 5.41 ± 0.5 µM and 4.08 ± 0.7 µM, respectively (Iacopetta et al. 2017). Zhao et al. evaluated vosaroxin, a type II DNA topoisomerase (Topo II) inhibitor, in HeLa cells and found it impaired cell survival, induced apoptosis via caspase-3 activation, oxidative stress, mitochondrial dysfunction, and decreased adenosine triphosphate (ATP) levels. Vosaroxin also inhibited hypoxia-inducible factor 1-alpha (HIF-1α) and activated the AMP-activated protein kinase (AMPK)/Sirtuin 3 (Sirt3) pathway, highlighting its potential as a cervical cancer chemotherapeutic agent (Zhao and Yu 2018). Sathish et al. synthesized β-carboline–podophyllotoxin conjugates from 4β-aminopodophyllotoxin and β-carboline acids to enhance cytotoxicity and inhibit type II DNA topoisomerase (Topo II). Compounds 10a and 10b induced S- and G2/M-phase cell-cycle arrest, acted as external DNA binders, and catalytically inhibited Topo II, showing strong cytotoxicity against HeLa cells with IC₅₀ values of 2.92 ± 1.12 μM and 3.72 ± 0.37 μM, respectively (Sathish et al. 2018). Shrestha et al. studied phenolic indenopyridinones and found that meta- and para-phenolic groups contributed to strong type II DNA topoisomerase alpha (Topo IIα) inhibition and antiproliferative activity. In HeLa cells, di-hydroxylated derivatives compounds 11 and 12 showed IC₅₀ values of more than 50 μM, and compounds 13, 14, 15 showed 2.25 ± 0.10 μM, 2.30 ± 0.47 μM, 4.32 ± 0.03 μM outperforming etoposide (IC₅₀ = 9.21 µM). Compounds (16, 17) showed IC₅₀ values of 4.72, 2.24, and 2.25 µM, while 2,4-diphenyl indenopyridinols (18 and 19) showed IC₅₀ values of 6.91 µM and 7.08 µM, demonstrating significant cytotoxicity (Shrestha et al. 2018). Reddy et al. (2018) synthesized podophyllotoxin derivatives with 4β-amidotriazole to enhance potency, selectivity, solubility, and pharmacokinetics. Compounds 20a and 20b induced G2/M-phase cell-cycle arrest and strongly inhibited type II DNA topoisomerase (Topo II) in HeLa cells, with IC₅₀ values of 2.47 ± 0.24 and 0.78 ± 0.02 μM, respectively (Reddy et al. 2018). Sangpheak et al. (2019) synthesized chalcone derivatives 21a–21c via the Claisen–Schmidt condensation to target human DNA topoisomerase II alpha (hTopo IIα). Chalcone 21b showed strong cytotoxicity and inhibited hTopo IIα-ATPase, with an IC₅₀ of 3.2 µM, highlighting its potential as an anticancer agent (Sangpheak et al. 2019). Singh et al. (2020) synthesized naphthalimide–1H-phenanthro[9,10-d] imidazole conjugates via Suzuki–Miyaura cross-coupling. Compound 22 showed DNA intercalation and type II DNA topoisomerase (Topo II) inhibition, exhibiting moderate cytotoxicity against HeLa cells with an IC₅₀ of 28.34 ± 0.22 μM (Singh et al. 2020). Fathy et al. (2020) studied a ciprofloxacin derivative, a 4-fluoroquinolone antibiotic, which inhibited mammalian type II DNA topoisomerase (Topo II) and showed strong anticancer effects in HeLa cells. The compound induced apoptosis via caspase-3 activation and Bax upregulation, reduced proliferation and colony formation, inhibited migration, and displayed anti-metastatic potential, highlighting its promise for cervical cancer therapy (Fathy et al. 2020). Kunwar et al. synthesized 2,4-diphenyl-5H-indeno[1,2-b]pyridinols with fluorophenyl and phenolic groups, showing strong type II DNA topoisomerase alpha (Topo IIα) inhibition. Compound 23 exhibited potent antiproliferative activity against HeLa cells with an IC₅₀ of 4.77 ± 0.028 μM (Kunwar et al. 2021). Magar et al. (2021) developed benzofuro[3,2-b] pyridin-7-ol derivatives via a one-pot, eco-friendly synthesis. Hydroxyl and halogen substitutions enhanced DNA topoisomerase IIα (Topo IIα) inhibition and drug–receptor binding, with compound 24 showing strong cytotoxicity against HeLa cells (IC₅₀ = 4.02 μM) (Magar et al. 2021). Song et al. (2022) synthesized xanthone derivatives and evaluated their cytotoxicity and topoisomerase inhibition. Compounds 25a and 25b showed notable HeLa cell cytotoxicity (IC₅₀ = 11.04 ± 0.56 μM and 8.904 ± 0.26 μM) via selective type II DNA topoisomerase (Topo II) inhibition, highlighting their potential as Topo II-specific anticancer agents (Song et al. 2022). Table 5 represents the list of Topo II inhibitors in pre-clinical studies.

**Table 5:** Cytotoxicity and enzyme inhibition profile of TOPO II inhibitors

TOPO II Inhibitors	MTT assay IC_50_ values	Enzyme inhibition/Drug Concentration	Cell lines	References
	2.19 ± 0.70 μM	60.1% at 100 μM	HeLa	(Cho et al. 2010)
	5.41 ± 0.5 μM	10 μM	HeLa	(Iacopetta et al. 2017)
	4.08 ± 0.7 μM	10 μM	HeLa	(Iacopetta et al. 2017)
	2.92 ± 1.12 μM	10 μM	HeLa	(Sathish et al. 2018)
.	3.72 ± 0.37 μM	10 μM	HeLa	(Sathish et al. 2018)
.	> 50 μM	68.9% at 100 and 20.8% at 20 μM	HeLa	(Shrestha et al. 2018)
	> 50 μM	100% at 100 μM and 93.7% at 20 μM	HeLa	(Shrestha et al. 2018)
	2.25 ± 0.10 μM	23.3% at 100 μM	HeLa	(Shrestha et al. 2018)
	2.30 ± 0.47 μM	72.8% at 100 μM and 12.0% at 20 μM	HeLa	(Shrestha et al. 2018)
	4.32 ± 0.03 μM	71.9% at 100 μM and 60.5% at 20 μM	HeLa	(Shrestha et al. 2018)
	4.72 ± 0.17 μM	100% at 100 μM and 30.5% at 20 μM	HeLa	(Shrestha et al. 2018)
	2.24 ± 0.02 μM	9.3% at 100 μM	HeLa	(Shrestha et al. 2018)
	6.91 μM	55.1% at 100 μM and 17.8% at 20 μM	HeLa	(Shrestha et al. 2018)
	7.08 μM	60.9% at 100 μM and 26.7% at 20 μM	HeLa	(Shrestha et al. 2018)
	0.78 ± 0.02 μM	100 μM	HeLa	(Reddy et al. 2018)
	3.2 μM	7.5 nM	HeLa	(Sangpheak et al. 2019)
	28.34 ± 0.022 μM	50 μM,100 μM	HeLa	(Singh et al. 2020)
	4.77 ± 0.028 μM	5.4% at100 μM	HeLa	(Kunwar et al. 2021)
	4.02 μM ± 0.01	93.1% at 100 μM and 26.5% at 20 μM	HeLa	(Magar et al. 2021)
	11.04 ± 0.56 μM	100 μM	HeLa	(Song et al. 2022)
	8.904 ± 0.26 μM	100 μM	HeLa	(Song et al. 2022)

These studies highlight the importance of type II DNA topoisomerase (Topo II)–targeting scaffolds in cervical cancer therapy, inducing DNA damage, cell-cycle arrest, and apoptosis. Among them, benzoxanthone (7), carbazole (9), β-carboline–podophyllotoxin conjugates (10a, 10b), indenopyridinones (11–19), podophyllotoxin–amidotriazole derivatives (20b), chalcone (21b), 2,4-diphenyl-5H-indeno[1,2-b]pyridinol (23), benzofuro[3,2-b]pyridin-7-ol (24), and xanthone (25b) showed the most potent Topo II inhibition and cytotoxicity, making them promising candidates for further optimization and in vivo evaluation.

### Topoisomerase II inhibitors in clinical trials

In research conducted by Pfeiffer et al., 32 patients with advanced or recurrent cervical cancer received intravenous teniposide as a single agent at a dosage of 100 mg/m^2^ on days 1 through 3 every three weeks. None of the patients had previously received cytotoxic treatment. Seven out of the thirty-two participants were able to provide a partial response, and none of them were able to provide a complete response, yielding a response rate of 22% overall. Furthermore, the illness advanced in 12 individuals (37%) and showed no change in 13 patients (41%). Leukopenia was the most frequent side effect, and the level of toxicity was considerable. In addition to minor nausea and vomiting, thrombocytopenia and leukopenia of WHO grade 3 or 4 were seen in some individuals. Although this study suggests that teniposide has limited efficacy in cervical cancer, its minimal toxicity, it holds potential for integration with chemotherapy in managing advanced or recurrent cancer like cervical (Pfeiffer et al. 1990). In research by Calero et al., tolerability and efficacy of the chemotherapeutic drug epirubicin were evaluated in a Phase II medical study involving thirty subjects confirmed with cervical squamous cell carcinoma.There were two full replies and three partial responses out of the 27 evaluable patients, or an 18.5% response rate. In the case of each subject undergoing therapy, the midpoint survival duration amounted to eight months. Importantly, the therapeutic agent was found to be generally tolerated, with negligible hematological adverse effects observed, and no instances of World Health Organization grade four toxic events occurred. The study found that individuals with cervix squamous cell carcinoma respond significantly to epirubicin at a dosage of 80 mg/m^2^. Given the good response rate of 18.5% and the absence of significant bone marrow toxicity, administration of higher doses of epirubicin may be considered (Calero et al. 1991). Table 6 represents the Topo II inhibitors in clinical trials.

**Table 6:** Topo II inhibitors in clinical trails

Cancer type	Drugs (dose)	Number of evaluable patients	Complete response	Partial response	Response rate	Reference
Advanced or recurrent cervical cancer	Teniposide (100 mg/m^2^)	32	-	22%	22%	(Pfeiffer et al. 1990)
Cervical squamous cell carcinoma	Epirubicin (80 mg/m^2^)	27	2	3	18.5%	(Calero et al. 1991)

#### Limitations of Topo II inhibitors in clinical trials

**Fig. 3:**
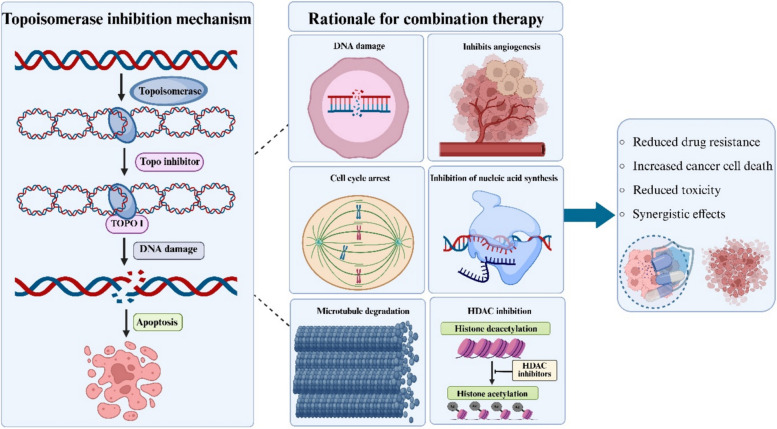
Representation of rationale of combining topoisomerase inhibitors with other chemotherapeutic agents. Combining topoisomerase inhibitors with other therapeutic agents enhances anticancer efficacy by targeting multiple cellular pathways simultaneously. While topoisomerase inhibitors induce DNA damage, co-administered agents can promote cell cycle arrest, inhibit nucleic acid synthesis, and trigger microtubule degradation, thereby strengthening cytotoxic effects. Additionally, drugs with HDAC inhibitory activity enhance chromatin accessibility and apoptosis, and anti-angiogenic effects further limit tumor growth (Created using BioRender)

The studies are limited by small patient cohorts and the use of single-agent therapy, which restricts efficacy in advanced or recurrent cervical cancer. Neither teniposide nor epirubicin produced high response rates or durable complete responses, indicating limited standalone activity. Teniposide was associated with considerable hematologic toxicity, while epirubicin showed only modest efficacy despite good tolerability. Collectively, these findings indicate that while single-agent efficacy is limited, their favorable toxicity profiles make them valuable candidates for combination regimens or optimized therapeutic strategies to improve outcomes in advanced cervical cancer. Fig. 3 represents the rationale of combining topoisomerase inhibitors with other therapeutic agents.

### Combination therapy in pre-clinical studies

Larasati et al. (2011) studied naringenin, a citrus flavonoid, and doxorubicin in HeLa cells. Individually, their IC₅₀ values were 195 µM and 1 µM, respectively, while combined treatment (100 µM naringenin + 0.5 µM doxorubicin) showed synergistic cytotoxicity, inducing S-phase arrest, sub-G1 accumulation, increased Bax, and decreased Bcl-2, indicating apoptosis (Larasati et al. 2011). Zeng et al. (2013) studied sanazole and irinotecan in hypoxic HeLa cells. Irinotecan (3 μM) alone or combined with sanazole (1 mM) reduced cell viability, induced G2-phase arrest, and enhanced radiosensitivity, demonstrating a significant synergistic radiosensitizing effect (Zeng et al. 2013). The combination of doxorubicin (DOX), a type II DNA topoisomerase inhibitor, and vorinostat (VOR), a histone deacetylase inhibitor, showed synergistic cytotoxicity in HeLa cells. Treatment (0.2 μM DOX + 2.5 μM VOR) induced apoptosis, DNA damage, nuclear deformation, and caspase-3 activation, with Bad upregulation in p53-proficient cells, suggesting enhanced efficacy at lower DOX doses (Lee et al. 2014). Panobinostat had a synergistic impact that increased cervical cancer cells' apoptosis when combined with topoisomerase inhibitors. Several dosages of etoposide (0–100 μM), topotecan (0–10 μM), and panobinostat (0–1000 nM) in cell viability assay for 24, 48, and 72 h to observe the cytotoxic effects, and the caspase-3/7 activity in both cell lines is markedly increased in comparison to when the drugs are administered separately (Wasim And Chopra 2016). Yuniarti et al. (2018) studied 1,2-epoxy-3(3-(3,4-dimethoxyphenyl)−4H-1-benzopyran-4-on) propane (EPI), a clove leaf oil derivative, in cervical cancer cells. IC₅₀ values were EPI was 33.24 μg/ml, doxorubicin was 23.34 μg/ml, and cisplatin was 4.8 μg/ml. EPI showed cytotoxicity and synergistic effects with doxorubicin and cisplatin, increasing expression of p53, TIMP-3, and miR-34a, suggesting its potential as a combination therapy (Yuniarti et al. 2018). Thymoquinone combined with etoposide in HeLa cells exhibited dose-dependent cytotoxic and genotoxic effects. The IC₅₀ of etoposide was 167.3 µM at 24 h and 52.7 µM at 48 h, while thymoquinone showed IC₅₀ values of 233.6 µM at 24 h and 145.5 µM at 48 h. At higher concentrations, thymoquinone enhanced etoposide’s cytotoxicity and DNA-damaging effects, whereas at lower concentrations it reduced DNA damage, indicating its potential to improve etoposide’s anticancer efficacy (Nur Çelebioğlu et al. 2022).

These studies show that combining natural compounds or epigenetic modulators with chemotherapeutics enhances apoptosis, DNA damage, and cell-cycle arrest in cervical cancer cells. Combinations like naringenin, EPI, thymoquinone, or panobinostat with doxorubicin, cisplatin, or etoposide improve efficacy, reduce doses, and offer promising cervical cancer therapies.

### Combination therapy in clinical trials

Papadimitriou evaluated the MVAC regimen (Vinblastine, Doxorubicin, Methotrexate, Cisplatin) in 27 metastatic cervical cancer patients. Objective responses were seen in 52% of patients (11% complete, 41% partial), with median overall survival of 11 months and progression-free survival of 8 months among responders. Common toxicities included alopecia, neutropenia, stomatitis, and nausea, which were partly mitigated by granulocyte-colony stimulating factor. MVAC demonstrates promising efficacy in metastatic cervical cancer (Papadimitriou et al. 1997). Lissoni et al. evaluated the CEP regimen (Cisplatin, Epirubicin, Paclitaxel) in 49 patients: 30 with endometrial adenocarcinoma and 19 with cervical adenocarcinoma. In endometrial cancer, therapeutic and histopathological responses were 73% and 35%, while in cervical cancer, responses were 64% and 62%, the response rate was denoted to be 53%. The study highlights CEP’s strong anticancer activity and tolerability, supporting further controlled trials to confirm its efficacy as first-line therapy (Lissoni et al. 1997). Umesaki et al. studied the MEP regimen (Mitomycin C, Etoposide, Cisplatin) in 31 patients with stage IVb or recurrent cervical cancer. Overall, 16.1% responded, including 4 complete and 1 partial remission, with higher response (26.7%) in chemotherapy-naïve patients (Umesaki et al. 1999). Sugiyama et al. evaluated cisplatin plus irinotecan (CPT-11) in 29 patients with advanced or recurrent cervical cancer. The overall response rate was 59% (52% partial, 7% complete). Dose-limiting toxicities included leukopenia, neutropenia, and diarrhea, with neutropenia being most severe, confirming the regimen’s efficacy despite myelosuppression (Sugiyama et al. 2000). D’Agostino et al. studied neoadjuvant chemotherapy with paclitaxel, epirubicin, and cisplatin in 42 patients with regionally advanced cervical cancer. Among 33 evaluable patients, the overall response rate was 78.5% (8 complete, 25 partial), with 25 patients responding before surgery. Main toxicities included hematologic effects, vomiting, nausea, and alopecia. The study concluded that this regimen, followed by radical surgery, is effective and feasible despite notable toxicity (D'Agostino et al. 2002). Dunton et al., aimed to establish the highest safely tolerated dosage of the drug topotecan when given alongside external beam radiotherapy for late-stage cervical carcinoma. Therapy for 9 subjects diagnosed with cervical squamous cell carcinoma began with topotecan at 0.5 mg/m^2^, followed by dose escalation in 0.25 mg/m^2^ steps. According to the study found that topotecan, at a dosage of 1.0 mg/m^2^, may be safely provided during external-beam radiation treatment for advanced cervical cancer. The overall response rate was reported to be 28%. (Dunton et al. 2002). Machida et al. conducted a Phase I study on CPT-11 and nedaplatin in 12 patients, establishing recommended doses of 50 mg/m^2^ and 60 mg/m^2^, respectively. Among 8 evaluable patients, the combination showed a 50% response rate, with 2 complete and 2 partial responses, though some experienced dose-limiting toxicity (Machida et al. 2003). Tsuda et al. (2004) studied irinotecan (CPT-11) combined with nedaplatin (254-S) in 27 patients with advanced or recurrent cervical cancer. The maximum tolerated doses were 60 mg/m^2^ for CPT-11 and 80 mg/m^2^ for nedaplatin, with recommended doses of 50 mg/m^2^ and 80 mg/m^2^, respectively. The combination achieved a 59% overall response rate (2 complete, 14 partial), with median survival of 415 days and median progression-free survival of 161 days, demonstrating clinical efficacy (Tsuda et al. 2004). Le et al. conducted a Phase II multicenter trial evaluating carboplatin plus pegylated liposomal doxorubicin (PLD) in 53 patients with recurrent or metastatic cervical or uterine cancer (51 evaluable). The overall response rate was 33%, with six-month progression-free survival of 43% and overall survival of 86%. Common grade 3–4 toxicities included fatigue, nausea, vomiting, and dyspnea, but the combination was generally well tolerated (Lê et al. 2005). Fabbro et al. (2010) conducted a Phase I study in 15 patients with locally advanced cervical cancer to determine optimal weekly doses of irinotecan and cisplatin with pelvic radiotherapy. Fixed cisplatin (20 mg/m^2^) and irinotecan (30 mg/m^2^) were given with weekly irradiation, and the recommended dose for Phase II trials was established as cisplatin 20 mg/m^2^ and irinotecan 35 mg/m^2^ (Fabbro et al. 2010). Ying et al. (2011) studied neoadjuvant chemotherapy (NACT) with irinotecan hydrochloride and cisplatin in locally advanced cervical cancer. Patients received two cycles of NACT before surgery, while the control group underwent surgery alone. NACT reduced pelvic lymph node metastases, deep stromal invasion, and positive surgical margins, lowered intra-pelvic recurrence, and improved overall survival and two-year relapse-free survival (Ying et al. 2011). Manci et al. (2011) In locally advanced cervical cancer (IB2–IIIB), neoadjuvant chemotherapy (NACT) with cisplatin (75 mg/m^2^, day 1) and topotecan (0.75 mg/m^2^/day, days 1–3) showed promising efficacy and manageable toxicity. Among 46 patients, two-year progression-free survival (PFS) and overall survival (OS) were 70% and 81%, respectively; in 38 operated patients, PFS and OS were 79% and 95%. The regimen achieved 15.8% complete response and 89.5% objective pathologic response, supporting its feasibility for further trials (Manci et al. 2011). Watanabe et al. showed that recurrent cervical cancer can be treated safely with oral etoposide and intravenous cisplatin. The study reported a median progression-free survival of 4.5 months, overall survival of 9.7 months, and an overall response rate of 16.7%, indicating the regimen’s feasibility (Watanabe et al. 2011). Kurtz et al. evaluated carboplatin plus oral topotecan for advanced or recurrent squamous cervical carcinoma. The maximum tolerated topotecan dose was 3.0 mg/m^2^ with carboplatin AUC 5, but due to toxicity in 9 of 12 patients, the phase II trial was halted. Weekly oral topotecan with carboplatin was deemed unsafe, highlighting the need for alternative regimens (Kurtz et al. 2012). Ferrandina et al. studied 75 patients with locally advanced cervical cancer (LACC) using neoadjuvant chemotherapy (NACT) with paclitaxel, epirubicin, and cisplatin (TEP) followed by radical surgery. Among 73 evaluable patients, 13 achieved complete and 28 partial responses (ORR 56.1%). Three- and five-year overall survival rates were 55% and 51%, respectively. Grade 3–4 leukopenia and neutropenia occurred in 29.7% and 30.9% of patients. TEP showed good efficacy and operability with manageable toxicity (Ferrandina et al. 2013). Zighelboim et al. studied topotecan, cisplatin, and bevacizumab in patients with relapsed or persistent cervical cancer. Administered every 21 days, the regimen showed a six-month progression-free survival of 59%, with both partial and complete responses. Among 26 patients, 1 complete response (4%) and 8 partial responses (31%) and the overall response rate was 35%. Positron emission tomography–computed tomography (PET/CT) was used to monitor early treatment response. Despite efficacy, high toxicity was observed, highlighting the need for biomarkers to predict response and optimize dosing (Zighelboim et al. 2013). Topotecan, used for recurrent cervical cancer, boosts cisplatin effectiveness by blocking DNA repair. In a 2016 multicenter Phase II study, Tsuda et al. assessed its safety and anticancer activity in previously treated uterine cervix squamous cell carcinoma patients. Encouraging results prompted the Gynecologic Oncology Group (GOG) to test the combination of topotecan and cisplatin in Phase III trials. The ORR was 28%, with 3 CR and 6 PR. (Tsuda et al. 2016). Table 7 represents the clinical trials of combination therapy.

**Table 7:** Clinical trials of combination therapy

Cancer type	Drugs (dose)	Number of evaluable patients	Complete response	Partial response	Response rate	Reference
Metastatic cervical carcinoma	Vinblastine(3 mg/m^2^) ^#^Doxorubicin(30 mg/m^2^) Methotrexate(30 mg/m^2^) Cisplatin(70 mg/m^2^)	27	11%	41%	40%	(Papadimitriou et al. 1997)
Cervical adenocarcinoma	Paclitaxel(175 mg/m^2^) ^#^Epirubicin(70 mg/m^2^) Cisplatin(50 mg/m^2^)	19	6%	-	53%	(Lissoni et al. 1997)
Recurrent cervical adenocarcinoma	^#^Etoposide(100 mg/m^2^) Cisplatin(50 mg/m^2^) Mitomycin C(10 mg/m^2^)	31	12.9%	3.2%	16.1%	(Umesaki et al. 1999)
Recurrent cervical carcinoma	Cisplatin(60 mg/m^2^) *Irinotecan(60 mg/m^2^)	29	7%	52%	59%	(Sugiyama et al. 2000)
Advanced cervical carcinoma	Cisplatin(100 mg/m^2^) ^#^Epirubicin(100 mg/m^2^) Paclitaxel(175 mg/m^2^)	42	19%	59.5%	78.5%	(D'Agostino et al. 2002)
Late-stage cervical carcinoma	*Topotecan (1 mg/m^2^) External Beam Radiation	9	-	-	28%	(Dunton et al. 2002)
Recurrent cervical carcinoma	*Irinotecan (50 mg/m^2^) Nedaplatin (60 mg/m^2^)	8	-	-	50%	(Machida et al. 2003)
Recurrent cervical carcinoma	*Irinotecan(50 mg/m^2^) Nedaplatin(80 mg/m^2^)	27	7%	52%	59%	(Tsuda et al. 2004)
Recurrent cervical carcinoma	Carboplatin (AUC 5) ^#^Doxorubicin(35 mg/m^2^)	51	6%	19%	33%	(Lê et al. 2005)
Advanced cervical carcinoma	Cisplatin(20 mg/m^2^) *Irinotecan(35 mg/m^2^)	15	43%	35%	78%	(Fabbro et al. 2010)
Advanced cervical carcinoma	Cisplatin(60 mg/m^2^) *Irinotecan(60 mg/m^2^)	60	-	-	65%	(Ying et al. 2011)
Advanced cervical carcinoma	Cisplatin (75 mg/m^2^) *Topotecan (0.75 mg/m^2^)	46	15.8%	73.7%	89.5%	(Manci et al. 2011)
Recurrent cervical carcinoma	Cisplatin(50 mg/m^2^) ^#^Etoposide(25 mg/day)	25	-	-	16.7%	(Watanabe et al. 2011)
Recurrent squamous cell carcinoma	*Topotecan(3 mg/m^2^) Carboplatin (AUC 5)	18	-	25%	28%	(Kurtz et al. 2012)
Locally advanced cervical carcinoma	Cisplatin(100 mg/m^2^) ^#^Epirubicin(100 mg/m^2^) Paclitaxel(175 mg/m^2^)	73	17.8%	38.3%	56.1%	(Ferrandina et al. 2013)
Persistent cervical carcinoma	*Topotecan(0.75 mg/m^2^) Cisplatin(50 mg/m^2^) Bevacizumab(15 mg/kg)	26	4%	31%	35%	(Zighelboim et al. 2013)
Recurrent cervical carcinoma	Cisplatin (50 mg/m^2^) *Topotecan (0.75 mg/m^2^)	32	-	-	28%	(Tsuda et al. 2016)

^*^Topo I inhibitor, # Topo II inhibitor

Among the studies, CEP (Cisplatin, Epirubicin, Paclitaxel), TEP (Paclitaxel, Epirubicin, Cisplatin), and Topotecan plus Cisplatin showed the best balance of efficacy and tolerability in cervical cancer patients, making them the most promising regimens for further clinical research. MVAC was effective but limited by toxicity, while Cisplatin plus Irinotecan showed potential with optimized dosing. Notably, Topotecan plus Cisplatin demonstrated enhanced efficacy in recurrent disease, leading to Phase III evaluation by the Gynecologic Oncology Group (GOG).

### Translational gaps

Although extensive preclinical and clinical studies support the therapeutic rationale for topoisomerase inhibitors in cervical cancer, their clinical benefit, particularly in advanced or recurrent cervical cancer has remained inconsistent. This discrepancy highlights a persistent translational gap between experimental efficacy and patient outcomes. Drug candidates may show efficacy in cell models but fail in in vivo or humans due to poor safety, insufficient target engagement, inadequate pharmacokinetics or bio availability, lack of superiority over existing therapies. Additional barriers include limitations of preclinical models, incomplete understanding of disease biology and difficulties in intriguing integrating complex datasets (Seyhan 2019). Additionally biological factors such as persistent expression of high-risk human papillomavirus (HPV) oncoproteins E5, E6 and E7 leads to sustained inactivation of p53 and retinoblastoma (Rb) pathways, resulting in defective cell cycle regulation, impaired DNA damage response, and genomic instability (Moody And Laimins 2010).

Topoisomerase inhibiting chemotherapeutic agents should be administered selectively to patients most likely to benefit from them. The use of ineffective regimes can delay appropriate therapy, promote the development of drug resistance, and cause unnecessary toxicity. However, identifying reliable predictors of response to topoisomerase inhibitors remains challenging due to the presence of multiple redundant DNA repair pathways that enable cancer cells survival despite topoisomerase induced damage (Pommier 2013).

Pharmacological limitations are another reason for translational failure. For example, SN-38 exhibits strong preclinical activity including p53/p21 activation and apoptosis induction; however, its poor solubility and unfavourable for pharmacokinetics precluded clinical use as a standalone agent. Although irinotecan improved drug delivery, it's high variable metabolomic conversion to SN38 (approximately 2 to 8%) in patients resulted in unpredictable exposure and inconsistent therapeutic benefit these pharmacokinetic constraints are particularly limiting in heavily pretreated or recurrent cervical cancer populations, where tumour heterogeneity and reduced treatment tolerance further compromise efficacy (Yu et al. 2023).

Despite strong preclinical efficacy of the camptothecin analog CDK-602 (belotecan) which exhibited apoptosis, G2/M arrest, and tumour regression, these effects failed to translate clinically. In a phase II trial, belotecan, monotherapy showed no objective responses in recurrent or progressive cervical cancer and caused mainly haematologic toxicity the median overall survival of 12.38 months highlights the limited efficacy emphasising the gap between preclinical and clinical efficacy (Lee et al. 2019; Hwang et al. 2011).

### Cutting edge approaches in cervical cancer therapy

#### Nanoformulations

DNA topoisomerases are critical anti-cancer targets, but clinical translation of their inhibitors is restricted by dose-limiting toxicities. Conventional therapies are limited by low specificity, systematic toxicity, and drug resistance, prompting the exploration of nanotechnology-based drug delivery systems. Nanocarriers including liposomes, polymeric, carbon-based, inorganic, and protein-based nanoparticles enhance tumour targeting, drug stability, bioavailability, and reduce of target toxicity (Hegde et al. 2023).

Preclinical studies demonstrate the efficacy of several nanocarrier platforms: lipid-coated 3D graphene oxide nanoparticles co-delivering doxorubicin and topotecan showed increased cytotoxicity in HeLa cells compared with free drugs (Nandi et al. 2019). Doxorubicin-loaded gold nanoparticles induced mitochondrial depolarization and apoptosis (Akinyelu et al. 2022). Carvacrol loaded nanoparticles enhance the efficacy of topoisomerase inhibitors at lower doses with the DOX–carvacrol combination showing synergistic effects (Akhlaq et al. 2023). Silver nanoparticle bound carboplatin stabilise DNA topoisomerase complex causing increased DNA damage and apoptosis at lower concentrations (Venkatas and Singh 2020). Hyaluronic acid-chitosan nanoparticles delivering doxycycline selectively targeted CD-44 expressing cervical cancer cells, providing pH-responsive controlled release, high encapsulation efficiency and minimal toxicity to normal cells (Anjum et al. 2025). Additional platforms, such as dual stimuli-sensitive ferrosoferric oxide (Fe_3_O_4_) magnetic nanoparticle incorporated nano hydrogels also demonstrated controlled drug release and significant cytotoxicity against HeLa cells under magnetic heating (Gupta And Gupta 2017).

Nano formulated topoisomerase inhibitors have shown modest efficacy in clinical trials for recurrent or previously treated cervical cancer. Biweekly liposomal doxorubicin (Lipo-Dox, 20 mg/m^2^) demonstrated limited clinical benefit, with disease progression in 90% of patients and only one partial response. Median survival was two months, and toxicity were mostly mild to moderate (Chen et al. 2008). Pegylated liposomal doxorubicin administered every four weeks produced partial responses in 11.1% of patients, with generally acceptable tolerability (Rose et al. 2006). Combined therapy with carboplatin and liposomal doxorubicin achieved a modest overall response rate of 38% and median survival of 37 weeks, though myelosuppression and infusion-related reactions were notable (Verschraegen et al. 2001).

#### Biomarker guided therapy

Biomarker guided patient selection is critical for improving cervical cancer treatment outcomes due to the disease biological heterogeneity and HPV driven pathogenesis. Established biomarkers such as HPV DNA, p16 over expression and squamous cell carcinoma antigen (SCC-Ag), along with the emerging markers including VEGF (vascular endothelial growth factor) and cancer stem cells markers (Nanog, SOX2, ALDH1) and circular RNAs, offer opportunities for improved patient stratification and therapeutic optimization (Purohit et al. 2025). Mechanic stoically, HPV-mediated disruption of the G1/S checkpoint render cervical cancer cells highly dependent on G2/M checkpoint following DNA damage. Topoisomerase I inhibitors induce DNA strand breaks marked by γ-H2AX, while activation of the ATR–Chk1–Wee1 pathway facilitates DNA repair and therapeutic resistance. Elevated γ-H2AX, Wee1, and Chk1 expression correlates with poor chemotherapy response, supporting their clinical utility as predictive biomarkers and providing a rationale for combining topoisomerase inhibitors with G2/M checkpoint inhibitors (Vici et al. 2016).

#### Antibody drug conjugates (ADCs)

Antibody drug conjugates (ADCs) are targeted biopharmaceuticals that link monoclonal antibodies to potent cytotoxic agents via chemical linkers by selectively delivering cytotoxins to tumour cells through antigen recognition, ADCs increase anti-tumour efficacy while minimising off target toxicity, addressing key limitations of conventional chemotherapy and improving the therapeutic potential of antibody-based treatments (Wang et al. 2025).

Sacituzumab govitecan (IMMU‑132) is a Trop‑2 (trophoblast cell surface antigen 2) –targeted ADC that delivers SN‑38, a potent TOP1 inhibitor, to Trop‑2–expressing cervical cancer cells. In a non‑randomized, open‑label Phase II study (NCT05838521), patients with recurrent or persistent cervical cancer received sacituzumab govitecan after progression on prior systemic therapy. The study enrolled patients with measurable disease and evaluated objective response rate (ORR) as the primary endpoint, with secondary endpoints including progression‑free survival (PFS), overall survival (OS), duration of response, and safety/tolerability. Treatment was generally well tolerated, with adverse events consistent with the known safety profile of sacituzumab govitecan (https://clinicaltrials.gov/study/NCT05838521n.d.).

Tisotumab vedotin, a tissue factor–targeted ADC, demonstrated superior efficacy compared with chemotherapy, with higher objective response rates, longer median PFS, and improved OS, including in patients previously treated with immunotherapy. Trastuzumab deruxtecan (TDxd) has also shown notable activity in HER2 (Human epidermal growth factor receptor 2) overexpressing cervical cancers, although HER2 positive tumors represent a small fraction of cases. Another Trop 2–targeted ADC, sacituzumab tirumotecan, is currently under investigation in a Phase 3 trial as a second or third-line therapy for recurrent or metastatic cervical cancer (An et al. 2025). These studies highlight the potential of ADCs as tumour selective, potent therapies in cervical cancer.

## Discussion

Cervical cancer remains a major global health burden, particularly in low-resource countries, with high morbidity and mortality rates, and current treatments show limited efficacy in advanced disease with significant toxicity, underscoring the urgent need for improved therapeutic strategies (Burmeister et al. 2022). Topoisomerases are essential for DNA replication and repair, making them attractive anticancer targets for decades. Topo I and II inhibitors are integral to chemotherapy due to their broad-spectrum antitumor activity (Yakkala et al. 2023). These agents induce DNA damage and apoptosis by stabilizing topoisomerase–DNA cleavage complexes and activating caspases, while also contributing to chromatin fragmentation during programmed cell death (Sordet And Solier 2012).

Pre-clinical studies demonstrate that structurally diverse Topo I inhibitors such as piperazine-substituted 3-arylisoquinolinamine, hydantoin, β-carboline–bisindoles, and chromenoquinoline derivatives (Van et al. 2014; Majumdar et al. 2015; Kovvuri et al. 2018; Zhou et al. 2022), along with Topo II targeting scaffolds including benzoxanthone, carbazole, β-carboline–podophyllotoxin conjugates, indenopyridinones, chalcone, and xanthone derivatives (Cho et al. 2010; Iacopetta et al. 2017; Sathish et al. 2018; Shrestha et al. 2018; Sangpheak et al. 2019; Song et al. 2022), exhibit significant anticancer activity. Their planar frameworks promote DNA intercalation and cleavage-complex stabilization, supporting rational scaffold optimization. Additionally, combinations of naringenin, EPI, thymoquinone, or panobinostat with doxorubicin, cisplatin, or etoposide enhance apoptosis and DNA damage, highlighting synergistic and potentially less toxic therapeutic strategies in cervical cancer (Larasati et al. 2011; Wasim And Chopra 2016; Yuniarti et al. 2018; Nur Çelebioğlu et al. 2022).

Clinical studies of Topo I and II inhibitors as monotherapies in cervical cancer are constrained by small cohorts and limited efficacy in advanced disease. Topotecan and belotecan showed modest benefit with notable toxicity (Muderspach et al. 2001; Hwang et al. 2011), while teniposide and epirubicin (Pfeiffer et al. 1990; Calero et al. 1991) demonstrated low response rates and limited durability, with teniposide causing significant hematologic toxicity and epirubicin offering only modest efficacy. In clinical studies topoisomerase inhibitors often show limited efficacy and notable toxicity as monotherapies, prompting exploration of combination strategies. Topotecan plus cisplatin demonstrated improved activity and advanced to Phase III evaluation by the GOG (Tsuda et al. 2016), yet many empirically designed combinations have provided only modest benefit with considerable hematologic toxicity.

The development of multitarget agents represents a promising strategy, as topoisomerase inhibitors have the potential to interfere with mechanisms driving tumor resistance and progression in recurrent cervical cancer (Fu et al. 2017). Novel approaches, including nanoformulations (Hegde et al. 2023), biomarker-guided therapies (Purohit et al. 2025), and ADCs (Wang et al. 2025), aim to address the limitations of conventional topoisomerase-based treatments. Nanocarriers enhance tumor targeting and drug stability while reducing systemic toxicity, although their clinical benefits remain modest. Biomarker-driven strategies tackle the biological heterogeneity of HPV-associated cervical cancer, enabling rational patient stratification and combination approaches targeting DNA damage response pathways. Importantly, ADCs delivering Topo I inhibitors, such as sacituzumab govitecan and tisotumab vedotin (An et al. 2025; https://clinicaltrials.gov/study/NCT05838521n.d.), represent a major advancement by allowing selective and potent tumor cell killing with manageable toxicity. These strategies underscore shift towards precision-based tumour selective therapies to enhance efficacy and durability of response in cervical cancer.

## Data Availability

No datasets were generated or analysed during the current study.

## Abbreviations

ADCs: Antibody drug conjugates
AMPK: AMP-activated protein kinase
ATP: Adenosine Triphosphate
CENPF: Centromere Protein F
CEP: Cisplatin, Epirubicin, and Paclitaxel
CIS: Cisplatin
CPT: Camptothecin
CPT-11: Irinotecan
CTCF: CCCTC-binding factor
DOX: Doxorubicin
EPI: 1,2-Epoxy-3(3-(3,4-dimethoxyphenyl)-4H-1-benzopyran-4-on) propane
GOG: Gynecologic Oncology Group
HCPT: Hydroxycamptothecin
HDAC: Histone deacetylase
HIF-1α: Hypoxia Inducing Factor
HIV: Human immunodeficiency virus
HPV: Human Papilloma Virus
hTopIB: Human DNA topoisomerase IB
ICRF-159: Razoxane
ICRF-187: Dexrazoxane
LACC: Locally Advanced Cervical Cancer
LDH: Lactate Dehydrogenase
Lipo-Dox: Liposomal doxorubicin
MEP: Mitomycin C, Etoposide, and Cisplatin
miR-34a: MicroRNA 34a
MST-16: Sobuzoxane
MTD: Maximum Tolerable Dose
mtDNA: Mitochondrial DNA
MVAC: Vinblastine, Doxorubicin, Methotrexate, and Cisplatin
NACT: Neoadjuvant chemotherapy
ORR: Objective response rate
OS: Overall Survival
PARP1: Poly (ADP-ribose) polymerase -1
PET/CT: Positron emission tomography–computed tomography
PFS: Progression-free survival
PLD: Pegylated Liposomal Doxorubicin
Rb: Retinoblastoma
ROS: Reactive Oxygen Species
RS: Radical Surgery
SCC-Ag: Squamous cell carcinoma antigen
Sirt3: Sirtuin 3
TDP1: Tyrosyl-DNA Phosphodiesterase 1
TDxd: Trastuzumab deruxtecan
TEP: Paclitaxel, Epirubicin, Cisplatin
TIMP-3: Tissue inhibitor of metalloproteinases-3
Topo I: Topoisomerase I
Topo II: Topoisomerase II
Topo IIα: Topoisomerase IIα
Topo IV: Topoisomerase IV
Tyr723: Tyrosine residue
URR: Upstream regulatory region
VEGF: Vascular endothelial growth factors
VOR: Vorinostat
WHO: World Health Organization
